# Utilization of Netnography as a Health Care Research Methodology: Scoping Review

**DOI:** 10.2196/78025

**Published:** 2025-10-24

**Authors:** Amany Sadat, Elizabeth Green, Imogen Forsythe, Stacey Munnelly, Georgette Eaton, Matthew Wynn, Fiona Pearson, Emma Dobson

**Affiliations:** 1 School of Health and Society University of Salford Salford United Kingdom; 2 Faculty of Medical Sciences, The Catalyst, Science Square, Newcastle Helix NIHR Innovation Observatory, Population Health Sciences Institute Newcastle University Newcastle upon Tyne United Kingdom; 3 Manchester University NHS Foundation Trust Manchester United Kingdom; 4 Nuffield Department of Primary Care Health Sciences University of Oxford Oxford United Kingdom; 5 London Ambulance Service NHS Trust London United Kingdom; 6 School of Nursing and Advanced Practice Liverpool John Moores University Liverpool United Kingdom

**Keywords:** health care, netnography, research methods, scoping review

## Abstract

**Background:**

Netnography is an emergent qualitative methodology adapted from ethnography to explore interactions and cultural dynamics within digital environments. Although it is increasingly used in health care research, its application remains inconsistent, particularly regarding methodological transparency and ethical reporting. Given netnography’s growing use in health care and the limited guidance on its application, a timely review of how it is defined and operationalized in the literature is warranted.

**Objective:**

This scoping review aims to identify, examine, and report how netnography has been defined and operationalized in the health care literature.

**Methods:**

A scoping review was conducted in accordance with the Joanna Briggs Institute framework and reported following PRISMA-ScR (Preferred Reporting Items for Systematic Reviews and Meta-Analyses extension for Scoping Reviews) guidelines. Comprehensive searches across 20 databases and gray literature sources identified peer-reviewed and academic studies that used netnography or netnographic methods within health care. Records were independently double-screened against prespecified eligibility criteria informed by the National Institute for Health and Care Excellence topic classifications. Data from the included studies were charted and synthesized narratively to generate the findings.

**Results:**

Eighty-two studies were included, spanning diverse health care topics, populations, and digital platforms. Netnography was most frequently applied to explore health communication, chronic illness, patient empowerment, and health care experiences, particularly among stigmatized or hard-to-reach groups. Ethical transparency varied widely: only 33 studies reported obtaining formal ethical approval, and just over half addressed informed consent.

**Conclusions:**

Netnography holds significant promise for health care research, offering insights into lived experiences and access to otherwise inaccessible populations. However, inconsistent methodological and ethical reporting raises concerns about rigor and accountability. To strengthen future applications, clearer guidance is needed on ethical standards, methodological justification, and reporting practices, particularly when researching vulnerable groups and sensitive health issues.

## Introduction

### Background

Netnography, developed by Robert Kozinets in the 1990s, is an immersive, observational research method designed to explore “technoculture”—the identities, behaviors, and communities emerging within online environments [1,2]. In this review, “netnography” refers specifically to the structured qualitative approach defined by Kozinets [2], which involves the systematic observation and analysis of naturally occurring interactions in online health communities, rather than broader online ethnographic or trace-based methods. Adapted from ethnography, it focuses on interpreting meaning in online interactions across platforms such as blogs, forums (eg, Mumsnet), social networks (eg, Facebook), content communities (eg, TikTok, Instagram), and virtual environments (eg, World of Warcraft, Second Life) [3]. As the first ethnographic method tailored for online spaces [4], netnography extends traditional ethnography by conveying social stories [5] and examining the lived experiences of individuals in digital environments [6]. While netnography has found widespread application in marketing, sociology, and anthropology, its use in health care research remains limited, fragmented, and inconsistently reported [7]. This gap motivates the present review, which seeks to clarify how netnography has been applied in health care contexts.

In health care, netnography can provide meaningful insights into patient behaviors, treatment preferences, health care pathways, and unmet needs by examining informal, peer-led online communities where individuals with shared health concerns support one another [8]. Previous applications include studies of chronic illness peer support, such as in Parkinson disease [5], public attitudes toward vaccination, and the exchange of coping strategies for mental health [9]. However, there has been no comprehensive synthesis of how netnography has been adapted and operationalized in health care, nor of the methodological challenges this presents.

Applying netnography in health care raises questions about the validity and generalizability of data derived from digitally engaged populations, as online users may communicate differently than they do in clinical settings. Issues of authenticity, identity fluidity, and the interpretive nature of digital contexts also present challenges for meaningful analysis and may limit the applicability of findings in clinical or policy contexts [1,3,4]. Although these discussions may lack professional input, they offer valuable opportunities to understand patient perspectives and inform health care innovation [10].

### Theoretical Framework

This review is grounded in broader theoretical debates on digital epistemology and participatory culture, which clarify how netnography both extends and challenges traditional qualitative methods in health research. Digital epistemology, as explored by Lupton [11] and Floridi [12], examines how knowledge is produced and legitimized online, particularly through user-generated and peer-shared content. Participatory culture, as theorized by Jenkins and Ito [13], describes the collaborative, networked nature of online engagement, which is increasingly relevant in patient communities. Aligned with social constructivist paradigms, this review views health meanings and identities as co-constructed through digital interaction [14]. As such, netnography serves both as a method and as a lens for understanding how patients share, negotiate, and embody health knowledge online. While Kozinets laid the methodological foundation, scholars such as Pink et al [6] have expanded netnography’s scope. Lupton [11] emphasized digital embodiment and affect, urging attention to more than just textual data, while Pink et al [6] highlighted the importance of sensory experience, materiality, and the embodied nature of online interactions. Together, these perspectives situate netnography within the evolving discourse of digital ethnography, particularly relevant in complex, data-rich health contexts. These theoretical insights informed the operationalization of this scoping review by guiding how we mapped the adaptation of netnography in health care, the types of knowledge it produces in digital contexts, and the methodological challenges reported across studies. Despite its growing recognition in fields such as nursing [10], netnography remains underreported and underutilized in health care research.

A scoping review was therefore selected to map this emerging area, as it enables synthesis across heterogeneous definitions and methodological approaches [15], aligning with current methodological recommendations [15]. Unlike systematic reviews, which address narrowly focused questions supported by relatively homogeneous evidence, scoping reviews are better suited to broad, exploratory questions and diverse study designs [16,17].

### Review Question

The review of this scoping review aimed to answer:

How and why is netnography utilized as a research methodology within health and care research?

### Aims and Objectives

This scoping review aims to identify, examine, and report how netnography is defined and operationalized in the health care literature, offering guidance for future studies and helping to assess the sufficiency of current evidence to inform subsequent systematic reviews [18]. The study objectives are as follows:

- Map and describe the key characteristics of studies applying netnography in health care contexts, including populations, settings, and study aims.
- Critically examine how netnography is defined and conceptualized, noting variations in terminology and theoretical framing across studies.
- Evaluate the methodological operationalization of netnographic approaches, identifying patterns, inconsistencies, and adaptations in study design and execution.
- Investigate the ethical considerations reported in the conduct of netnographic research, with particular attention to consent, privacy, and researcher positionality.
- Assess the justifications provided for using netnography and the extent to which it is positioned as an appropriate or necessary approach for addressing health care research questions.
- Identify conceptual, ethical, and methodological gaps to inform future applications of netnography in health and care research.

## Methods

### Protocol and Registration

This scoping review was conducted in accordance with a registered protocol on the Open Science Framework (OSF) registries [19]. It followed the Joanna Briggs Institute methodology, a widely recognized and rigorous framework for conducting scoping reviews [20]. Reporting of this review is guided by the PRISMA-ScR (Preferred Reporting Items for Systematic Reviews and Meta-Analyses extension for Scoping Reviews) guidelines. A completed PRISMA-ScR checklist, indicating the page numbers where each reporting item is addressed, is provided in Multimedia Appendix 1.

### Eligibility Criteria

To be eligible for inclusion, studies were required to have employed netnographic methods exploring the perspectives, experiences, or behaviors of individuals within any health care population (eg, patients, practitioners) in any health care setting or service. The scope of eligible studies was further refined using the National Institute for Health and Care Excellence (NICE) topic classifications (Multimedia Appendix 2) to ensure alignment with practical health care domains and support relevance for clinical policy translation. While this approach enhances applicability, it may limit the inclusion of studies focused on broader social or behavioral health topics. NICE provides a well-established framework covering core clinical and public health domains. Although this UK-specific focus may limit direct generalizability to other health systems, it offers a structured lens for examining how netnography is applied across key health care areas.

**Figure 1:**
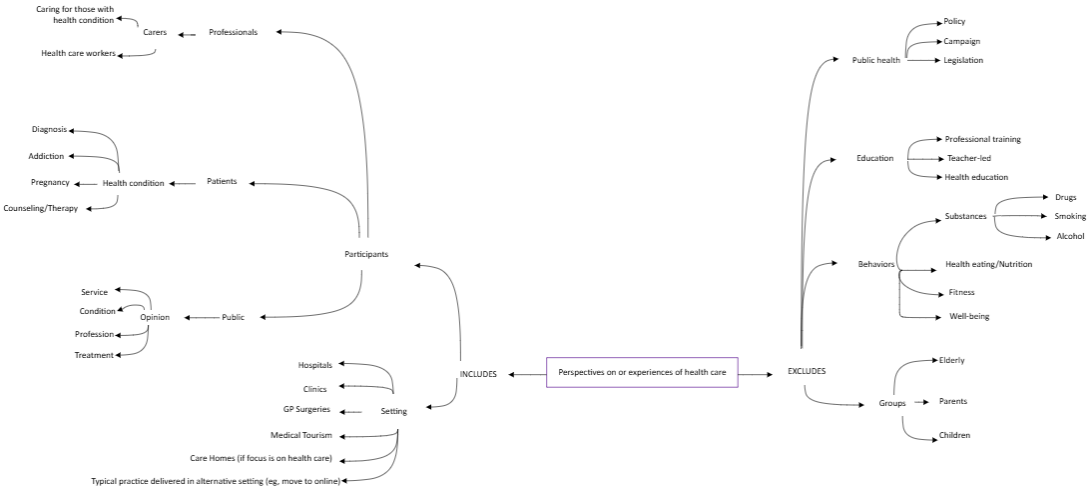
Diagram of eligibility criteria with reference to the perspectives and experiences of health care being examined through a netnographic approach.

Eligible studies were empirical, employed netnographic methods as explicitly identified by the original authors, examined perceptions or experiences related to health care, and were published in English (see Figure 1).

Synonyms such as “online ethnography” were deliberately excluded to focus specifically on netnography, a distinct methodological approach designed for studying online communities with greater rigor compared with broader ethnographic methods.

Table 1 provides a summary of the inclusion and exclusion criteria.

**Table 1:** Inclusion and exclusion criteria applied at both the title and abstract and full-text screening stages.

Summary	Inclusion criteria	Exclusion criteria
Population	Studies undertaken by those working within or researching health care; focusing on using netnography to explore the experiences or perspectives of any health care professional, health care administrator (involved in service design, commission, delivery, or audit), patient or recipient of health care products or services, or members of the public irrespective of age, sex, gender, ethnicity, and sociodemographic background	Studies led by researchers from non–health care disciplines (eg, anthropology, sociology, education) with aims unrelated to understanding health care experiences. For example, netnographies on health care teaching methods or health product marketing were excluded.
Concept	Qualitative, quantitative, or mixed-method studies that explicitly adopted a netnographic approach as defined by the authors and stated in the title, abstract, or methods section.	Literature reviews, conceptual and theoretical articles
Context	Research conducted in any health care setting or covering any digital health topics within the narrowed scope of NICE^a^ classifications.	Research not aligned with health care topics as defined by NICE classifications; studies based in public health or behavior change
Limits	English language	Non-English language

^a^NICE: National Institute for Health and Care Excellence.

### Information Sources

Databases searched were Ovid (APA PsycArticles, Embase, MEDLINE, Health Management Information Consortium [HMIC], Ovid Journals), Web of Science (Core Collection, ProQuest Dissertations & Theses, KCI, CSCD, SciELO), ProQuest (PTSDpubs, Social Sciences Collection), EBSCO (CINAHL, Child Development & Adolescent Studies), ScienceDirect, Scopus, PubMed, JSTOR, and the VHL Regional Portal. All databases were searched in January 2024, with no restrictions on publication year (see Textbox 1). Search results were exported to EndNote (version X21; Clarivate Analytics), and duplicates were removed by an information specialist (ED). To complement database searches, gray literature was identified from relevant websites, including The King’s Fund Digital Archive, Social Care Online, Nuffield Trust, Africa Research Database, 3ie Development Evidence Portal, CORE, NDLTD Global ETD Search, and Google Scholar. Google Scholar searches were conducted in incognito mode to minimize the influence of personalized search histories. Following guidance from Haddaway et al [21], the first 200 Google Scholar results were screened for suitability. Reference lists of key reviews and included studies were also screened to identify additional relevant literature.

**Full list of searched databases.**

1. **Ovid**
  - APA PsycArticles
  - Embase
  - MEDLINE
  - Health Management Information Consortium (HMIC)
  - Ovid Journals
2. **Web of Science**
  - Web of Science Core Collection
  - ProQuest Dissertations & Theses
  - KCI Korean Journal Database
  - Chinese Science Citation Database
  - SciELO Citation Index
3. **ProQuest**
  - Coronavirus Database
  - PTSDpubs
  - Publicly available content
  - Social Sciences Premium Collection
  - Social Science Database
  - Sociology Collection
4. **EBSCO**
  - Child Development & Adolescent Studies
  - CINAHL
  - Bibliography of Asian Studies
5. ScienceDirect
6. Scopus
7. JSTOR
8. VHL Regional Portal

### Search Strategy

An initial search of CINAHL, MEDLINE, Embase, PsycArticles, PubMed, Scopus, and ProQuest Sociology was conducted to help refine a comprehensive search strategy. Titles, abstracts, and index terms were analyzed to optimize terminology, which was then applied across all selected databases. Broad terms such as “netnography,” “netnographic,” and “netnograph” were used to ensure inclusivity, without field-specific keywords, enabling the examination of netnography across diverse health care contexts. This approach was informed by the novelty of the methodology and inconsistent indexing [15]. Synonyms such as “online ethnography” were excluded due to limited indexing. The search strategy was peer reviewed by an information specialist using the PRESS (Peer Review of Electronic Search Strategies) guideline [22]. Further details are provided in Multimedia Appendix 3.

### Selection of Evidence for Inclusion

To ensure consistency in decision-making among reviewers, multiple consensus-checking points were implemented. First, 5 studies were screened to assess alignment in the application of eligibility criteria, informing the development of an “elaboration document” with examples (Multimedia Appendix 4). Each reviewer then independently screened 25 titles and abstracts [23]. Discrepancies were resolved through discussion, and the criteria were further refined. Citations were collated in EndNote, duplicates were removed, and the remaining citations were imported into Covidence (Veritas Health Innovation) for screening [23].

All full-text articles were independently and blindly screened by AS, ED, MW, SM, GE, EG, and IF. Any discrepancies (eg, when 1 reviewer included an article and another excluded it) were resolved by a third reviewer from among the authors to ensure consistent application of the inclusion criteria. In the next stage, full-text articles of potentially relevant studies were assessed during a pilot phase. Once agreement exceeded 75%, blinded full-text screening commenced, with 2 or more reviewers independently assessing each article. Reasons for exclusion were recorded.

### Data Charting Process

A data charting form was developed in Covidence to extract key study variables, including definitions of netnography, rationale, aims, health topics, data sources, participant demographics, methods, and ethical considerations. This form was initially piloted and then refined through consensus meetings before full data extraction was undertaken (see Multimedia Appendix 5).

### Data Items

Data were extracted on study and methodological characteristics and charted in Excel 2016 (Microsoft Corporation). Extracted items included study aims, netnography definitions, researcher positionality, methods, and ethical considerations. When positionality was not stated, it was recorded as “unclear.” Full data for all 82 articles are provided in Multimedia Appendix 6.

### Synthesis of Results

**Figure 2:**
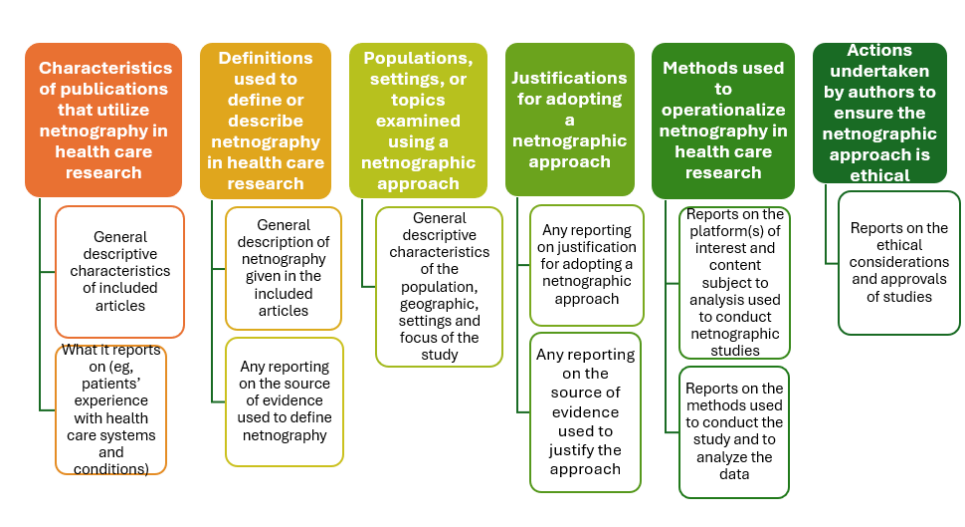
Utilization of netnography in health care research: scoping review data items extracted.

As outlined in our a priori plan, coding was guided by the data extraction form, with categories developed collaboratively by the review team. Data were initially synthesized using a descriptive approach, combining frequency counts with basic coding techniques to organize key patterns and identify gaps (Figure 2).

An inductive, iterative thematic analysis approach was applied, following the methodology of Braun and Clarke [24]. Initial codes were developed in Excel and then refined collaboratively through discussion and comparison across reviewers. Discrepancies in coding were resolved through consensus meetings. Themes and subthemes were reviewed and finalized using Covidence to ensure transparency and traceability.

## Results

### Selection of Sources of Evidence

**Figure 3:**
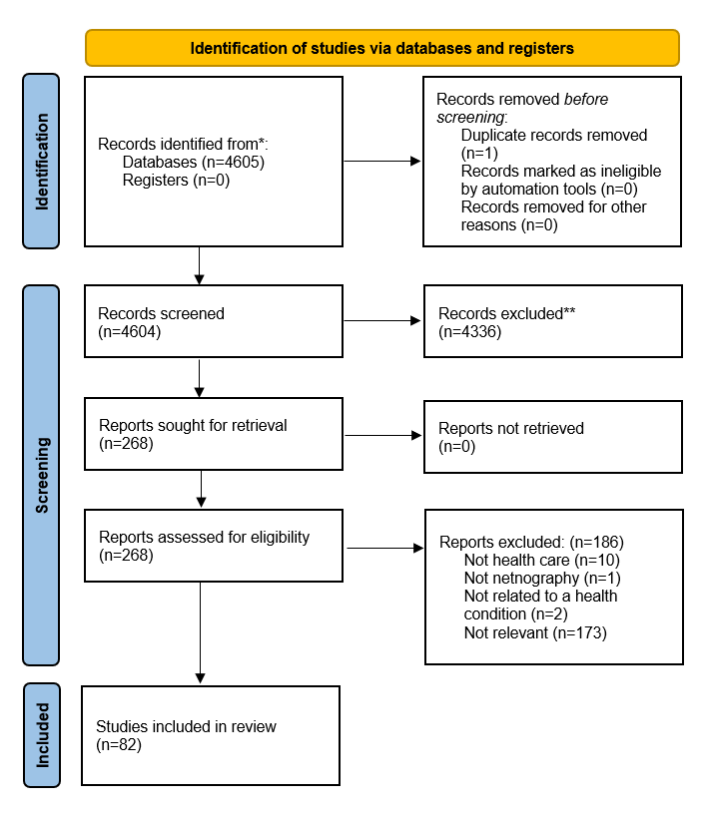
PRISMA (Preferred Reporting Items for Systematic Reviews and Meta-Analyses) flowchart. *Consider, if feasible to do so, reporting the number of records identified from each database or register searched (rather than the total number across all databases/registers). **If automation tools were used, indicate how many records were excluded by a human and how many were excluded by automation tools.

A total of 4605 records were identified through database searches. After the removal of 1 duplicate, 4604 records were screened by title and abstract. Of these, 4336 were excluded for not meeting the inclusion criteria. The remaining 268 articles were retrieved in full for eligibility assessment. After full-text screening, 186 studies were excluded, primarily for not being in health research areas (n=10), not using netnography (n=1), or not addressing a health condition (n=2). No studies were excluded due to retrieval issues. In total, 82 studies met the inclusion criteria and were included in the final review. This process is summarized in the PRISMA flowchart (Figure 3).

Data synthesis identified 5 primary themes: Social Media in Health Communication, Chronic Illness and Online Communities, Patient Empowerment, Health Care Experiences, and Family Networks in Digital Health.

### Characteristics of Sources of Evidence

**Figure 4:**
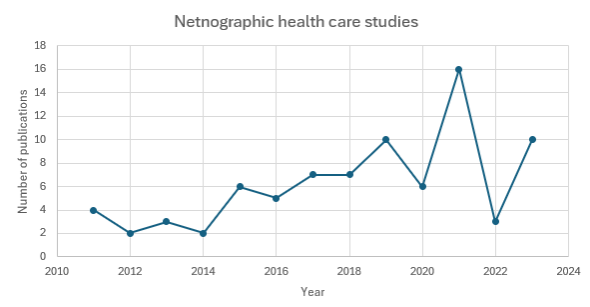
Number of articles adopting netnography in health care research.

Among the 82 included netnographic health care studies, the use of the methodology has steadily increased since 2011, with the majority published between 2019 and 2023 and peaking in 2021 (n=16; see Figure 4). Earlier years showed limited uptake. The sample comprised 68 journal articles, 8 dissertations [25-32], 3 conference papers [33-35], 2 book chapters [36,37], and 1 conference poster [38]. The increase since 2019 likely reflects growing recognition of online communities in health care research. It may also be related to accelerated digital engagement during the COVID-19 pandemic, highlighting netnography’s relevance for understanding patient behaviors in evolving digital contexts.

Of the 82 studies, 36 specified a geographic focus, while 46 did not. The most common location was the United Kingdom (n=10) [25,39-47], followed by the United States (n=7) [26,36,40,45,48-50]. Other countries were France (n=4) [5,41,46,51], Canada (n=3) [40,45,52], Brazil (n=3) [50,53,54], Turkey (n=3) [37,55,56], Germany (n=1) [41], Australia (n=3) [40,46,57], Italy (n=3) [41,46,58], Sweden (n=2) [59,60], Romania (n=1) [61], Spain (n=2) [41,46], Finland (n=1) [62], India (n=1) [63], Malaysia (n=1) [33], Poland (n=1) [64], Russia/Ukraine/Czech Republic (joint study) [65], Slovenia (n=1) [66], and New Zealand (n=1) [44]. These findings reflect the methodological adaptability of netnography across diverse settings.

### Results of Individual Sources of Evidence

#### Definitions and Conceptualizations of Netnography in Health Care Research

All 82 studies explicitly referenced netnography. One study mentioned it solely in the title [61]. Seven studies referenced netnography only within their abstract [34,37,40,47,67-69], and 15 studies mentioned it exclusively in the methods section [27,28,38,39,49,57,59,70-77]. Another study referenced netnography in both the title and abstract [78], while 20 studies referenced it in all 3 sections: title, abstract, and methods [5,29,33,42,44-46,50,54-56,63,78-84]. The placement of “netnography” varied across studies, suggesting differences in how central the method was to each study.

Fifty-nine studies provided a definition of netnography, while 23 did not. Among those offering definitions, 64 out of 82 (78%) cited Kozinets’ foundational work [9,25-37,39, 40,42-45,47-57,59-63,65-68,70,71,74,75,77,78,80,81,84-97], while others referenced Salzmann-Erikson and Eriksson [58,73,98-100] or additional authors such as Hine [92,101], Hookway [43,102], Bowler [50,103], and Krippendorff [96,104]. Most definitions described netnography as an ethnography-based qualitative method for exploring cultures, behaviors, and interactions in online environments. Although terminology varied, definitions generally emphasized its flexibility, context-sensitivity, and adaptation to the digital age.

#### Focus of Topics Areas

A key focus of the included studies was the role of social media in health communication. Twenty-five studies explored how users seek, share, and co-create health information online, thereby shaping collective health knowledge [5,9,25,26,28,30,33,35,44,61,62,64,72,73,75-77,88-90,95-97,105].

Thirty-three studies examined online communities supporting individuals with chronic conditions, highlighting the importance of digital platforms in developing peer connections, providing emotional support, and fostering community resilience [27,29,31,37-41,43,45,46,48,50,53-55,59, 67,69,70,74,78,79,81-83,87,91,92,98-100,106].

Six studies addressed patient empowerment and self-management, showing how individuals use online resources to guide health decisions and engage in care [36,47,57,68,80,93]. Ten studies explored health care experiences and how online interactions shape patient perceptions of care and systems [34,49,51,63,65,66,71,84-86]. These patterns suggest that netnography in health care primarily focuses on information sharing, peer support, and patient engagement in online spaces.

Five studies focused on family and close networks, examining how digital health advice and decisions are influenced within trusted social circles [50,56,58,60,94]. Most studies focused on patients (n=66) [5,6,9,26-29,31,32,34-36, 39-43,46-55,57,59,61,63-69,72-75,78-83,85-89,91-100,105,106], with others including carers (n=11) [5,38,42,46,54,60,63,75,77,80,94], parents (n=7) [50,56,58,63,77,92,94], the general public (n=11) [9,32,38,62,75-77,83,84,91,97], and practitioners (n=8) [25,42,44,47,57,71,77,84].

Several studies examined mixed groups (eg, patient-public, patient-practitioner). Forty-two studies did not clearly specify their population. Among defined groups, physical disabilities (n=13) [5,36,41,49,55,57-59,70,80,92,105,106], vulnerable populations (n=8) [9,32,61,62,64,75,96,99], children and young people (n=6) [56,57,92,98-100], and learning disabilities (n=5) [58,64,79,83,105] were commonly addressed. Other populations included carers, older adults, ethnic minorities, lesbian, gay, bisexual, transgender, and queer/questioning (LGBTQ+) individuals, and those at the end of life. The distribution of populations indicates that netnography predominantly focuses on patients while also encompassing diverse groups, highlighting its flexibility in exploring health experiences across different social and clinical contexts.

#### Justification of Netnographic Approach

Of the 82 included studies, 23 did not provide a rationale for using netnography. Among those that did, common justifications included the ability to explore sensitive or stigmatized topics (n=10), such as mental health, infertility, and trauma [39,45,48,61,65,75,87,89,98,99], and improved access to geographically dispersed or marginalized populations (n=9) [25,45,61,73,84,86,89,95,105]. Netnography was valued for its unobtrusiveness and reduced interaction bias (n=7), offering naturalistic, unfiltered insights into online behavior [28,35,59,86-88,96]. Some studies also cited its cost-effectiveness and efficiency (n=9) [28,35,51,56,59,71,86,88,96], while others praised its ability to generate rich, contextual data (n=6) [26,39,55,61,99,100] and its flexibility in exploring diverse or niche communities (n=9) [27,28,40,42,49,53,66,72,91]. Half of the studies (n=41) examined patient or caregiver experiences, often related to specific health conditions, treatments, or interactions with health care systems [5,9,26,27,29,31,32,39,40,43,45, 48-51,53-59,62,65,72,73,75,79,83,87,89,91,94-97,99,100,105,106]. These findings suggest that netnography is chosen for its unique ability to explore sensitive topics and reach hard-to-access populations, highlighting its value in capturing nuanced patient and caregiver experiences.

#### Data Sites Used

Among the 82 studies, researcher positionality varied: 19 adopted an active approach [29-33,42,47, 52,57,60,65,72,75,80,85,88-90,106], 37 a passive approach [9,26,28,34,36-41,45,48,50,51,56,58,59,61-64,69,70, 73,76-78,81,87,92,93,96,97,99,100,105], and the remaining 26 did not report positionality. Online forums were the most analyzed platform (n=33) [5,26,28,36,38,39,41,46-49, 51,52,55,58,60-62,67,69,72,73,79,81,88,90,92,95,96,98-100,105], followed by Facebook (n=17) [33,37,45,46,50,53,54,56,59, 64,69,77,78,82,88,89,106], Twitter/X (n=14) [30,31,38,41,46,48,54,69,77,78,80,88,89,94], YouTube (n=8) [9,50,54,73,75,76,87,91], and Instagram (n=4) [31,59,75,78]. Health-specific forums (eg, PatientsLikeMe) and blogs were rarely used, and 11 studies did not specify the platform. Most studies (n=40) focused on a single platform [9,25-28,30,33,37,42,44,50-53,56-58, 60-65,68,71,72,80-82,84,85,87,91,95-97,99,106], while a smaller number analyzed 2-5 platforms (n=17) [5,31,36,45,47,54,66,70,75-77,86,88,89,92,98,100], 6-10 platforms (n=9) [32,34,43,48,55,67,74,78,90], or more than 10 platforms (n=5) [40,49,69,93,105]. Platform numbers were not reported in 11 studies [29,35,38,39,41,46,59,73,79,83,94], indicating some inconsistency in methodological detail.

Content analyzed was mostly text-based (eg, posts, threads), with multimedia (eg, videos/images) included in a smaller subset. Most studies focused on a single content type (n=46) [5,9,25-27,29,30,32,36,39,40,45,50-52,55,56,60,61, 63-67,69-72,76,81,83-86,88,91-96,98-100,105], while 12 analyzed 2 content types [37,42,43,48,50,57,68,74,80,82,87,97], and 10 included 3 or more [44,47,49,53,54,58,59,62,73,75]. The remaining 14 studies did not specify content type.

Source volume ranged widely, with 30 studies analyzing over 1000 sources [26,30,31,40-43,45,46,49,52-55,57, 59,61,62,64,71,75,76,80,82,86-88,94-96]. A further 16 analyzed 101-500 sources [32,39,44,54,66,69, 70,72,74,78,84,85,92,94,99,100], 11 analyzed 501-1000 sources [26,37,48,51,56,58,61,67,90,97,105], and 10 reviewed under 100 sources [5,9,25,27,50,63,73,81,91,98]. However, the remaining 15 studies did not report source volume.

Thematic analysis was the most common analytic approach (n=43) [9,25,27,30,32,33,37,39-41,43-46,48-51,53,56, 58,60,64,67,69-72,74,76,78,81,82,84,86-88,92,94,97-100], followed by content analysis (n=13) [5,29,34,47,57,61,62,66,84,85,88,96]. Five studies used mixed methods [41,48-50,97], and 6 did not specify their analytic method [35,38,79,83,89,106]. The remaining 15 studies used various data analysis methods, including narrative analysis [80], grounded theory (n=4) [28,42,63,93], discourse analysis (n=2) [52,65], and the interpretive phenomenological approach [91]. These findings suggest that netnography in health care predominantly relies on text-based data and thematic analysis, reflecting its flexibility in handling different content types and volumes.

#### Approaches to Ethical Conduct in Netnography

Of the 82 studies, 51 reported that they sought ethical approval [5,25-28,30,31,37,39,40,42-46,48-54,56,58-61,65,67, 69,72-76,80-84,89,92-96,98-100,106], and approval was obtained in 33 of these [25,27,30,31,37,39,42,43,45,48-50, 52,56,58-61,72,74-76,80,81,89,92,94,96,98-100,106]. The remaining 31 studies did not report on ethical approval or provide related considerations, though 1 cited guidance suggesting that open forums may be used without consent [62]. Justifications for this included the use of publicly accessible data, institutional exemptions, or data unrelated to human participants (n=15) [5,26,28,40, 44,46,51,53,54,65,67,69,73,82,95]. Three studies provided no rationale [83,84,93].

Ethical practices were described in 48 studies, including anonymization of data [26,28,33,43,55,58,80,81,98], the use of pseudonyms, removal of identifying information, and efforts to minimize traceability. Some studies obtained proxy consent from organizations or informed users through digital channels. Four studies classified the content as publicly accessible, open-access online data (n=5) [48,51,54,76,93]. Researchers frequently referenced established ethical frameworks, including Kozinets [65,84], the Italian Psychological Association guidelines [58], and the Declaration of Helsinki [79]. One study justified the absence of formal ethical review by noting that, in the country of origin (Russia), no research ethics committee exists for this field [65].

Out of the 82 studies, informed consent was reported in 22 [25,29,31,37,42,52,56,57,59-61,64,72,74,82,83,87,89,92,96,99,106]. Of these, consent was obtained from content creators (n=12) [25,29,31,52,59-61,72,74,75,87,89], gatekeepers (n=6) [56,64,82,92,96,99], both content creators and gatekeepers (n=2) [57,106], interviewees (n=1) [83], or system administrators (n=1) [37].

Seventeen studies explicitly stated that informed consent was not obtained [26,28,30,33,45,46,50,51,65,67,75,76, 81,88,90,98,100], while 43 studies did not clarify whether consent was sought. Among the 60 studies that did not report obtaining informed consent, 31 nonetheless described ethical considerations and practices [5,26-28, 30,33,39-41,43,45,48,50,54,55,58,62,65,70,76,77,79-81, 84,88,93,95,98,100,105]. Ethical practices varied, but most studies took steps to protect privacy and ensure responsible conduct in online research, highlighting the importance of ethical vigilance in netnographic health care studies.

### Synthesis of Results

Netnography is increasingly used in health and care research to explore lived experiences, communication, and community dynamics within virtual communities. Typically applied as a qualitative, ethnographic method adapted for online contexts, it offers unobtrusive and cost-effective access to rich, contextual data. While ethical reporting and methodological transparency, particularly regarding consent and researcher roles, varied across studies, netnography was consistently valued for its flexibility and relevance to digital engagement. Researchers employed netnography to study naturally occurring, user-generated content on platforms such as forums, Facebook, and Twitter/X, especially in relation to sensitive health topics and hard-to-reach groups. Five key themes emerged: Social Media in Health Communication, Chronic Illness and Online Communities, Patient Empowerment, Health Care Experiences, and Family Networks in Digital Health.

These themes underscore netnography’s strength in capturing the everyday realities of digital health interactions.

We developed a typology to clarify conceptual variation in netnographic approaches. Table 2 summarizes the studies by type (pure vs hybrid) and level of researcher engagement (passive vs participatory), illustrating variation in methodological fidelity to Kozinets’ framework.

**Table 2:** Typology of netnographic engagement approaches in health care research.

Methodological fidelity	Passive researcher engagement (eg, observing forums without interaction)	Active researcher engagement (eg, posting, seeking consent, interacting)
Pure netnography (closely aligned with Kozinets’ framework [2])	Observing closed or open communities with fieldnote-like detail Strong reflexivity and immersion Clear ethical consideration	Full participation in online communities Disclosure of researcher identity Ethical copresence and prolonged engagement
Hybrid netnography (combined with other methods)	Using forum content alongside surveys or interviews Limited reflexivity or context Less immersion, but still observing real-world digital data	Using netnographic data as a supplement to ethnography or content analysis Researcher might engage in interviews or focus groups following online observation

## Discussion

### Summary of Evidence

Netnographic research in health care saw a notable rise, peaking in 2021 with 16 studies, likely driven by increased methodological awareness and the shift to digital engagement during COVID-19. Its ability to access natural, user-generated content makes it well-suited to capturing patient experiences, aligning with current health care research and policy priorities [107]. However, the review highlights inconsistent justification for using netnography. While some studies emphasized its relevance for sensitive or marginalized populations, others lacked a clear rationale, sometimes prioritizing accessibility over methodological fit. This raises concerns about its use for convenience, potentially compromising research rigor.

Ethical reporting was similarly inconsistent. While many studies addressed anonymity, core ethical principles such as autonomy and justice were often overlooked. Over half of the studies did not report informed consent, and ethical approval processes were described inconsistently. These gaps highlight ongoing challenges in digital research ethics, which could undermine participant protection and public trust. The persistence of ethical gaps in netnographic studies may stem from the tension between unobtrusive online observation, which allows access to authentic, naturally occurring discussions, and traditional research expectations of informed consent and participant protection [108]. Researchers often grapple with whether and when online data are truly “public” or if vulnerable online posters require enhanced protection, particularly given that community norms and platform privacy settings vary widely.

All 82 studies referenced netnography, though often in a technical rather than a theoretical context. This variation in reporting limits clarity and comparability across studies. These findings highlight netnography’s potential for exploring hard-to-reach populations while emphasizing the need for a more deliberate methodological and ethical approach. Researchers should ensure that netnography is purposefully chosen, particularly for sensitive topics, and consider how public awareness of data use might influence future online sharing. Finally, inconsistent reporting of data types, researcher roles, and analytic methods was a recurring issue across the included studies. This lack of transparency compromises the reproducibility of netnographic research and raises concerns about the trustworthiness of findings, which is particularly important in digital health contexts where methodological clarity underpins credibility. Ambiguity in researcher positionality, for instance, limits readers’ ability to assess how engagement shaped data interpretation, while vague analytic descriptions impede the evaluation or replication of results. As digital health research increasingly incorporates netnographic approaches, clearer and more consistent methodological reporting is essential for building a robust and reliable evidence base. Establishing standardized reporting guidelines for netnographic research in health care would enhance methodological rigor and promote best practices in this increasingly influential approach.

Researcher positionality and the degree of engagement in netnography significantly influence data validity and ethical considerations. Active participation can facilitate richer contextual insights and foster trust within online communities, but it may also introduce interaction biases. However, passive observation preserves the authenticity of naturally occurring data while raising ethical questions around consent and participant awareness. Understanding these dynamics is fundamental for evaluating netnographic fidelity and ensuring responsible research conduct.

Beyond describing researchers’ active or passive roles within online communities, positionality in qualitative research refers broadly to how researchers’ identities, experiences, beliefs, and disciplinary backgrounds influence multiple stages of the research process, including study design, data collection, interpretation, and the presentation of findings [8]. In netnography, particularly within sensitive health care contexts, positionality critically shapes how researchers engage with participants, obtain consent, interpret data, and make ethical decisions regarding representation and confidentiality.

Reflexivity, the ongoing practice of critically reflecting on one’s positionality and its influence, enhances transparency and rigor by making these influences explicit. Reflexivity strengthens ethical decision-making and deepens interpretation by helping researchers acknowledge potential biases and power dynamics inherent in the research relationship [109]. Recognizing positionality is therefore essential for transparent reporting and for navigating ethical responsibilities, ensuring a nuanced interpretation of sensitive online interactions.

To ensure methodological rigor and trustworthiness, we incorporated reflexivity throughout this review. Our review team recognizes our positionalities as researchers experienced in digital health, and we approached the ethical assessment with a conscious awareness of our potential biases. We critically reflected on challenges such as the limitations of consent mechanisms in online settings and the implications of ambiguous researcher roles. Regular team discussions enabled us to challenge and reflect on assumptions, helping to minimize bias in interpretation.

Our team combines diverse clinical, academic, and research expertise relevant to netnographic health care research. AS is an experienced nurse, lecturer, and doctoral student focusing on digital technologies. GE is a consultant paramedic who recently completed a DPhil (Oxon), applying netnography in her studies. SM is an advanced nurse practitioner and DProf student who also used netnography in her doctoral research. MW is a senior lecturer and nurse academic specializing in digital health. FP and ED are active researchers and evidence synthesis methodologists, while EG and IF specialize in evidence synthesis. These varied backgrounds enhanced our attentiveness to methodological rigor and ethical considerations in digital qualitative research. Throughout the review, our ongoing reflexive practice strengthened transparency and trustworthiness in the synthesis. When study aims were ambiguous or reporting on ethics was limited, our team’s interpretation and synthesis decisions were guided by our collective disciplinary and clinical perspectives.

While netnography offers distinct advantages in terms of accessibility, scalability, and cost-effectiveness, it also presents epistemological limitations. These include the risk of passive observation bias, where a lack of researcher engagement can lead to superficial interpretations; the decontextualization of online interactions, which may strip data of meaning; and challenges in verifying user identities, which can affect the reliability and validity of findings. These challenges highlight the need for reflexive, theory-informed approaches to strengthen the interpretive depth of netnographic research in health care. Variation in recruitment methods, consent practices, and analytic rigor across netnographic studies complicates the direct comparison and synthesis of findings. These inconsistencies may compromise the trustworthiness and reproducibility of netnographic research in health care, underscoring the need for clearer reporting standards and methodological transparency to strengthen the evidence base.

The findings reflect emerging practices in digital health communication and engage with ongoing debates around digital epistemology and participatory culture, as discussed by scholars such as Pink et al [6]. Health communication within online health care communities is shaped by the co-construction of knowledge among patients and professionals, aligning with constructivist paradigms [14] that emphasize the socially constructed nature of meaning on digital platforms. This theoretical perspective highlights how netnographic methods can reveal the dynamic interactions and participatory processes underlying digital health experiences, offering insights that go beyond descriptive accounts and contribute meaningfully to digital health research.

Our review extends previous syntheses of netnographic research, including Salzmann-Erikson and Eriksson’s [10] mapping review in nursing, which highlighted rising publication trends and recurring ethical challenges such as covert data collection and limited consent procedures. While their review focused specifically on nursing, our scoping review spans the broader health care landscape, incorporating diverse disciplines and conditions beyond nursing contexts.

Our review builds on previous findings by offering a cross-disciplinary synthesis that reveals inconsistent ethical reporting, variability in epistemological positioning, and limited reflexivity across health domains. Compared with discursive reflections such as Smith et al [110], which emphasize netnography’s adaptability in nursing, and systematic reviews in tourism [111], our review identifies unique methodological and ethical issues in health research, where sensitivity to vulnerable populations and clinical implications is critical. In addition, Delli Paoli and D’Auria’s [112] scoping review of digital ethnography highlights the diversity and fragmentation of netnographic approaches, including differences in data collection, ethical engagement, and contextualization. Our findings reinforce their call for methodological grounding and ethical nuance, particularly concerning covert research in sensitive health contexts.

By synthesizing applications across the health care spectrum, our review informs the development of field-specific standards for ethical and rigorous netnographic practice.

In reviewing the included studies, several recurring gaps emerged across topical focus, methodological approaches, ethical practices, and population coverage. These limitations may restrict the scope, credibility, and inclusivity of current netnographic health care research. Table 3 summarizes these gaps and outlines their implications for future research, highlighting opportunities to strengthen the ethical, methodological, and thematic breadth of digital health scholarship using netnography.

**Table 3:** Gap analyses.

Gap dimension	Specific gap identified	Implication for future research
Topics	Limited focus on underexplored areas such as mental health stigma, vaccine hesitancy, genetic testing, end-of-life care, and rare diseases.	Broaden topic coverage to include sensitive, complex, and socially nuanced health issues.
Methods	Sparse use of multimedia data (eg, images, video) and mixed-methods approaches.	Support diverse data types and analytic frameworks for richer findings.
Ethics	Inconsistent reporting on consent and a lack of clear ethical protocols.	Develop and adopt standardized ethical guidelines for netnography.
Population	Underrepresentation of older adults, LGBTQ+^a^ groups, ethnic minorities, and people at the end of life.	Prioritize inclusivity and diversity to improve health equity in digital health research.

^a^LGBTQ+: lesbian, gay, bisexual, transgender, and queer/questioning.

The review has identified ethical gaps in netnographic research, partly arising from institutional tensions. Traditional ethics principles—autonomy, beneficence, and justice [113]—were designed for face-to-face research and do not fully align with unobtrusive online observation. This creates uncertainty for researchers and ethics committees regarding how to apply these principles in digital contexts [114]. Many institutional review boards (IRBs) may underestimate the ethical significance of online interactions, often overlooking vulnerable participants. The rapidly evolving nature of digital platforms further complicates ethical governance. Moreover, labeling online content as “public” may conflict with users’ privacy expectations, underscoring the need for flexible, context-aware ethics frameworks. Bridging these gaps requires ongoing collaboration among researchers, ethics boards, and online communities to develop adaptive standards that both protect participants and support rigorous netnographic research.

Ethical inconsistencies were frequently reported across netnographic health studies, particularly concerning weak or absent consent procedures. Such gaps can undermine participant trust, especially in online settings where users may not expect to be studied, and they pose increased risks for marginalized or vulnerable groups susceptible to data misuse or exploitation. The absence of robust, context-sensitive consent models challenges the ethical integrity, credibility, and inclusiveness of netnographic health research. Informed consent is generally required when researchers directly engage with participants or use data from closed or private communities [115]. Nevertheless, ethical risks persist even in publicly accessible forums. Beninger et al [116] found that users often perceive their social media content as private, regardless of platform visibility. Researchers should, therefore, carefully consider contributor vulnerability, reidentification risks, and community norms before assuming that consent is unnecessary.

Given the fluid and anonymous nature of digital environments, fixed consent models are often insufficient. Flexible approaches—such as ongoing or dynamic consent that allow participants to update or withdraw permissions over time—are recommended to uphold participant autonomy throughout the research life cycle. Moreover, greater transparency regarding researcher involvement and clearer guidance from ethics committees or IRBs are essential, particularly concerning distinctions between public and private online data.

### Future Guidance

This review identifies significant variability in ethical reporting, including inconsistent consent practices, limited researcher reflexivity, and insufficient justification for data source choices. These issues risk participant harm, undermine trust, and reduce the credibility of digital health research, particularly for vulnerable populations. Ethical engagement in sensitive health communities should ideally involve transparent researcher presence (eg, disclosing one’s role in the group or profile), seeking gatekeeper or moderator approval, and anonymizing both data and platform names whenever possible. Lurking (ie, only observing) should be justified on a case-by-case basis, with careful attention to the potential for covert observation to harm trust or cause distress if later revealed. To address these challenges, we suggest conducting a follow-up study to develop practical tools and standards for ethical netnographic research in health care. This could include a decision matrix to guide ethical decision-making around data visibility, researcher interaction, and contextual risk, alongside standardized reporting frameworks to enhance transparency and methodological rigor. Such tools would support researchers, journals, and IRBs in promoting ethical and trustworthy netnographic research in digital health. Future netnographic research should combine ethical vigilance with methodological rigor. Researchers should clearly justify their data sources, analytic approaches, population inclusion, and ethical decisions. Practical tools, such as checklists or decision matrices, could guide choices regarding consent, researcher visibility, and data anonymization. Standardized reporting frameworks should document study design, sampling, analytic methods, and ethical steps to enhance transparency, reproducibility, and credibility. These measures will support ethical, inclusive, and methodologically robust netnographic research that meaningfully advances digital health scholarship.

### Limitations

This review has several limitations. It included only studies published in English with full-text availability, potentially excluding relevant research in other languages or without accessible full texts. Ethical approval, informed consent, and privacy practices were assessed based on self-reported data, which may not fully reflect actual practices. We also did not assess the professional backgrounds of study authors, such as affiliations with regulatory bodies, such as the Nursing and Midwifery Council or the Health and Care Professions Council, which could influence ethical conduct and reporting. Another limitation of this review is that it included only studies explicitly using the “netnography” label. This likely excludes research employing similar online ethnographic approaches under different terms, such as “digital ethnography” or “virtual ethnography,” which may affect the comprehensiveness of our findings. Our focus on netnography reflects its distinct methodological rigor, but future reviews could broaden search terms to capture related approaches and provide a more complete synthesis of digital qualitative work in health care. Future reviews could address this by expanding search strategies to include functional synonyms. Finally, inconsistencies in ethical and methodological reporting limit the ability to assess study rigor and introduce potential bias, underscoring the need for more transparent and standardized reporting in future netnographic health care research.

### Conclusions

This scoping review highlights the growing use of netnography in health care research, particularly for exploring patient experiences, digital health behaviors, and engagement within online communities. The method offers distinct advantages in accessing hard-to-reach populations and investigating sensitive health topics. However, the review identified inconsistent reporting of methodological and ethical practices, including limited justification for adopting netnography and variable attention to ethical approval and informed consent. Such gaps may compromise study transparency and rigor. To support the responsible and effective use of netnography in health care, clearer reporting standards and ethical guidance are needed. Future research should prioritize methodological justification, ethical reflexivity, and adherence to best practices to ensure the robustness and integrity of digital health research.

## Appendix

Multimedia Appendix 1 PRISMA-ScR (Preferred Reporting Items for Systematic reviews and Meta-Analyses extension for Scoping Reviews) checklist.

Multimedia Appendix 2 National Institute for Health and Care Excellence (NICE) topic classifications.

Multimedia Appendix 3 Search strategy and Peer Review of Electronic Search Strategies (PRESS) documentation.

Multimedia Appendix 4 Elaboration document.

Multimedia Appendix 5 Data extraction instrument.

Multimedia Appendix 6 Data items.

## Abbreviations

IRB: institutional review board
LGBTQ+: lesbian, gay, bisexual, transgender, and queer/questioning
NICE: National Institute for Health and Care Excellence
OSF: Open Science Framework
PRESS: Peer Review of Electronic Search Strategies
PRISMA-ScR: Preferred Reporting Items for Systematic Reviews and Meta-Analyses extension for Scoping Reviews
