# Supplementary figures and images for: Utilization of Netnography as a Health Care Research Methodology: Scoping Review
Source: J Med Internet Res. 2025 Oct 24;27:e78025. doi: 10.2196/78025 (PMC12595390; doi:10.2196/78025)

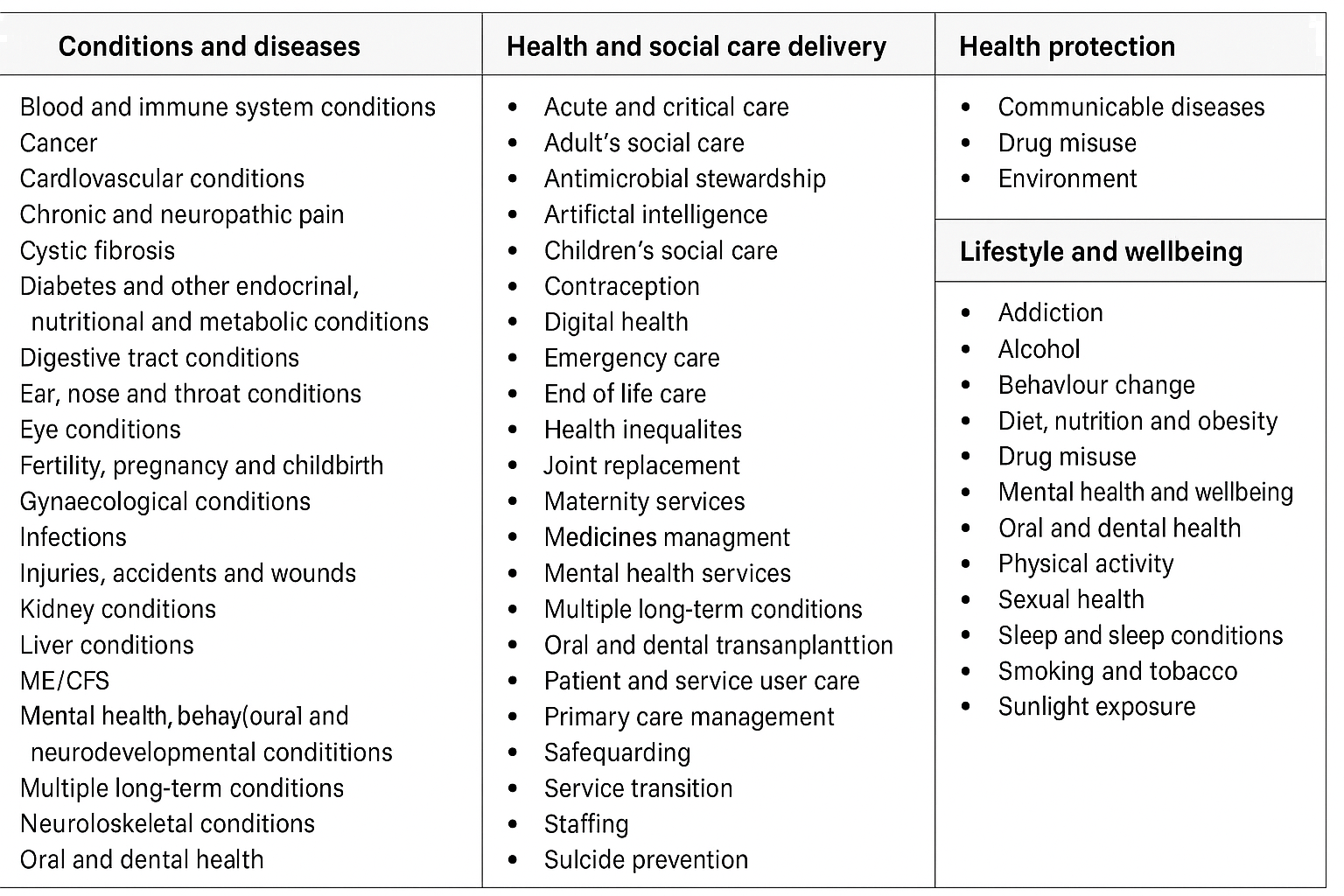

Supplement: Multimedia Appendix 2 [file jmir_v27i1e78025_app2.docx]
