# Supplementary material for: Utilization of Netnography as a Health Care Research Methodology: Scoping Review
Source: J Med Internet Res. 2025 Oct 24;27:e78025. doi: 10.2196/78025 (PMC12595390; doi:10.2196/78025)
Supplement: Multimedia Appendix 3 [file jmir_v27i1e78025_app3.docx]

| **EBSCO: CINAHL** | | |
| --- | --- | --- |
| *#* | *Search string* | *Records retrieved* |
| 1 | TI “netnography” | 20 |
| 2 | AB “netnography” | 44 |
| 3 | TI “netnographic” | 19 |
| 4 | AB “netnographic” | 34 |
| 5 | S1 OR S2 OR S3 OR S4 | 81 |
| 6 | S1 OR S2 OR S3 OR S4* | 78 |

Searched November 23, 2023, * limit to English language

**PRESS 2015 checklist for search strategy peer review**

| **Project name:** Netnography Trial Searches | | |
| --- | --- | --- |
| **Searcher :** Emma Dobson | **Checker:**  Claire Eastaugh | **Date:**  12/07/2024 |
| **Population** | - Netnography | |
| **Intervention** | - n/a | |
| **Comparator(s)** | - n/a | |
| **Outcome(s)** | - n/a | |
| **Search Strategy** | Appendix I | |
| **Databases searched** | \| Ovid \| APA PsycArticles Full Text \| \| --- \| --- \| \| APA PsycInfo (2002-2023) \| \| Embase (1996-2023) \| \| MEDLINE \| \| HMIC \| \| Ovid Journals \| \| WoS \| Core Collection \| \| MEDLINE \| \| Proquest Dissertations & Theses \| \| KCI - Korean Journal Database \| \| Chinese Science Citation Database \| \| Preprint Citation Index \| \| SciELO Citation Index \| \| Proquest \| Coronavirus Database \| \| PTSDPubs \| \| Natural Science Collection \| \| Publicly Available Content \| \| Social Science Premium Collection \| \| FirstSearch \| WorldCat \| \| WorldCatDissertations \| \| Proceedings First \| \| Papers First \| \| ArticleFirst \| \| EBSCO \| Child Development & Adolescent Studies \| \| CINAHL \| \| MEDLINE \| \| Bibliography of Asian Studies \| \| Elsevier \| ScienceDirect \| \|  \| \| Scopus \| Scopus \|  \| \| PubMed \| PubMed \|  \| \| JSTOR \| JSTOR \|  \| \| VHL \| VHL Regional Portal \|  \| \| Emerald \| Emerald Insight \|  \| \| GreyLit \| The King's Fund Digital Archive \|  \| \| SocialCareOnline \|  \| \| Nuffield Trust \|  \| \| Mednar \|  \| \| NDLTD Global EDTD Search \|  \| \| CORE \|  \| \| BASE \|  \| \| Africa Research Database \|  \| \| 3ie Development Evidence Portal \|  \| | |

| **Question** | **Y/N** | **Notes** |
| --- | --- | --- |
| **Translation of the research question** | | |
| Does the search strategy match the research question/PICO? | Yes |  |
| Are the search concepts clear? | Yes |  |
| Are there too many or too few PICO elements included? | Okay |  |
| Are the search concepts too narrow or too broad? | Okay |  |
| Does the search retrieve too many or too few records? (Please show number of hits per line.) | Okay |  |
| Are unconventional or complex strategies explained? | N/A |  |
| **Boolean and proximity operators (these vary based on search service)** | | |
| Are Boolean or proximity operators used correctly? | N/A |  |
| Is the use of nesting with brackets appropriate and effective for the search? | N/A |  |
| If NOT is used, is this likely to result in any unintended exclusions? | N/A |  |
| Could precision be improved by using proximity operators (eg, adjacent, near, within) or phrase searching instead of AND? | No |  |
| Is the width of proximity operators suitable (eg, might adj5 pick up more variants than adj2)? | N/A |  |
| **Subject headings (database specific)** | | |
| Are the subject headings relevant? | N/A |  |
| Are any relevant subject headings missing; for example, previous index terms? | No |  |
| Are any subject headings too broad or too narrow? | N/A |  |
| Are subject headings exploded where necessary and vice versa? | N/A |  |
| Are major headings (“starring” or restrict to focus) used? If so, is there adequate justification? | N/A |  |
| Are subheadings missing? | No |  |
| Are subheadings attached to subject headings? (Floating subheadings may be preferred.) | N/A |  |
| Are floating subheadings relevant and used appropriately? | N/A |  |
| Are both subject headings and terms in free text (see the following) used for each concept? | Yes | No subject headings used. No subject headings available. |
| **Text word searching (free text)** | | |
| Does the search include all spelling variants in free text (eg, UK vs. US spelling)? | N/A | No alternative spelling. |
| Does the search include all synonyms or antonyms (eg, opposites)? | N/A |  |
| Does the search capture relevant truncation (ie, is truncation at the correct place)? | Yes | Alternative suffixes possible, this is considered with the use of wildcard (*) search where available or individual keyword searches. |
| Is the truncation too broad or too narrow? | Okay | Netnograph* covers -y and -ic |
| Are acronyms or abbreviations used appropriately? Do they capture irrelevant material? Are the full terms also included? | N/A |  |
| Are the keywords specific enough or too broad? Are too many or too few keywords used? Are stop words used? | Okay | Keyword search for Netnography suitable for the search requirements. |
| Have the appropriate fields been searched; for example, is the choice of the text word fields (.tw.) or all fields (.af.) appropriate? Are there any other fields to be included or excluded (database specific)? | Yes | Relevant fields searched against the constraints and availability of the database. Search field .mp. where available; title, abstract and full text where available; all fields where available; topic where available. |
| Should any long strings be broken into several shorter search statements? | N/A | Single keyword search. No strings. |
| **Spelling, syntax, and line numbers** | | |
| Are there any spelling errors? | No |  |
| Are there any errors in system syntax; for example, the use of a truncation symbol from a different search interface? | No | Truncation by wildcard asterix (*) where available or full keyword search where necessary. |
| Are there incorrect line combinations or orphan lines (ie, lines that are not referred to in the final summation that could indicate an error in an AND or OR statement)? | N/A |  |
| **Limits and filters** | | |
| Are all limits and filters used appropriately and are they relevant given the research question? | N/A | No limits or restrictions imposed. |
| Are all limits and filters used appropriately and are they relevant for the database? | N/A |  |
| Are any potentially helpful limits or filters missing? Are the limits or filters too broad or too narrow? Can any limits or filters be added or taken away? | No | Would be inappropriate for the search to impose any. |
| Are sources cited for the filters used? | Yes | Clearly defined, search Portal and Databases clearly listed. |

| **Further comments:** |
| --- |
| For the requirements of the project, single keyword search is an appropriate strategy. Consideration has been made to conduct a broad search using the keyword “netnography”, appropriate truncation has taken place where the database allows, and the searcher has considered multiple suffixes. A very broad range of databases have been searched which will maximise the potential of finding relevant material. For the needs of the project ask, this is a robust search, clearly documented and reported. |
