# Supplementary material for: Utilization of Netnography as a Health Care Research Methodology: Scoping Review
Source: J Med Internet Res. 2025 Oct 24;27:e78025. doi: 10.2196/78025 (PMC12595390; doi:10.2196/78025)
Supplement: Multimedia Appendix 4 [file jmir_v27i1e78025_app4.docx]

|  |  |  |  |  |  |  |
| --- | --- | --- | --- | --- | --- | --- |
| **Is the study a netnography?**  The scoping review will consider empirical studies that have been identified by their authors as a netnography or adopting a netnographic approach to conduct research on health care. | | | | | | |
| **Is the study aim to improve understanding of a healthcare approach, condition or setting?** Research pertaining to any healthcare setting or topic that falls under the NICE topic classifications and that has been conducted by healthcare workers and/or researchers with the aims of furthering knowledge on perspectives or experiences of healthcare will be included. | | | | | | |
|  |  |  |  |  |  |  |
| Screening |  |  |  |  |  |  |
|  | *AS* | *SM* | *MW* | *ED* | *FP* | *GE* |
| Eriksson et al., (2013) | EXC | EXC | EXC | EXC | EXC |  |
| Salzmann-Erikson et al., (2016) | EXC | EXC | EXC | INC | INC |  |
| Roland et al., (2017) | EXC | EXC | EXC | EXC | EXC |  |
| Fernandes et al., (2023) | INC | INC | INC | INC | INC |  |
| Shahbazi et al., (2023) | EXC | EXC | EXC | INC | INC |  |
|  |  |  |  |  |  |  |
| Rationale |  |  |  |  |  |  |
| Eriksson et al., (2013) **EXCLUDE** | The aim of this paper is to 'describe communication about caring for infants among men who visited an Internet-based forum for fathers and elaborate on the dimensions of the support available on the forum'. Whilst this has links to child development, wellbeing and parental wellbeing, these links are implicit and not explicitly identified as an aim of netnographic investigation. As a result, this study would be excluded on context as it does not improve understanding of a health care approach, condition or setting. | | | | | |
| Salzmann-Erikson (2016) **INCLUDE** | The aim of this paper is to 'present culturally situated artefacts as depicted in nursing home environments and to analyse the underlying understandings of disciplining structures that are manifested in these kinds of places'. As care homes are identified as health care settings, this study would meet the context inclusion criteria as it helps to improve understanding of a health care setting and potential impact on workers and residents. | | | | | |
| Roland et al., (2017) **EXCLUDE** | The aim of this paper is to 'examine the emergence of a CoP through the study of social media interactions of the free open access medical (FOAM) movement'. Whilst the study examines interactions between physicians, nurses and paramedics, there is a focus on education rather than understanding an approach to support a health care condition or setting. | | | | | |
| Fernandes et al., (2023) **INCLUDE** | The aim of this paper is to 'understand how online communities can contribute to increasing the adherence of chronic patients to the treatment prescribed by the physician in Brazil', with a focus on participants posting on a Diabetes forum. As diabetes is a health condition, this study would meet the context inclusion criteria as it helps to improve understanding of patient experiences of health care. | | | | | |
| Shahbazi et al., (2023) **EXCLUDE** | The aim of this paper is to explore public health communication on social media during the Covid-19 pandemic. Covid-19 is a health condition (listed under infections in NICE guidance) but as the focus of the study is on public health communication rather than healthcare, this study would not meet the context inclusion criteria. | | | | | |
