# Supplementary material for: Utilization of Netnography as a Health Care Research Methodology: Scoping Review
Source: J Med Internet Res. 2025 Oct 24;27:e78025. doi: 10.2196/78025 (PMC12595390; doi:10.2196/78025)
Supplement: Multimedia Appendix 5 [file jmir_v27i1e78025_app5.docx]

| Source Citation |  |
| --- | --- |
| Source Type |  |
| Identified using the term ‘netnography’  (select all that apply) | In title  In abstract  In methods |
| Identified using the term ‘netnographic’  (select all that apply) | In title  In abstract  In methods |
| Facilitating platform(s) |  |
| Geographic focus  (if yes, please specify) | Y/N |
| Healthcare population of interest |  |
| Healthcare population age |  |
| Number of participants |  |
| Healthcare topic of interest |  |
| Healthcare setting of interest |  |
| Research questions/aims/objective |  |
| Definition of netnography |  |
| Justification for netnography |  |
| Researcher positionality | Active/Passive |
| Type of data collected |  |
| Method of data analysis |  |
| Ethical approval process reported | Y/N |
| Ethical justification (if authors did not seek ethical approval, do they include justification why, if yes please state) | Y/N/NA |
| Ethical considerations/actions reported |  |
| Informed consent | Y/N |
